# Membrane Remodeling by Arc/Arg3.1

**DOI:** 10.3389/fmolb.2021.630625

**Published:** 2021-03-08

**Authors:** Per Niklas Hedde, Leonel Malacrida, Barbara Barylko, Derk D. Binns, Joseph P. Albanesi, David M. Jameson

**Affiliations:** ^1^ Department of Cell and Molecular Biology, University of Hawaii at Manoa, Honolulu, HI, United States; ^2^ Laboratory for Fluorescence Dynamics, University of California, Irvine , CA, United States; ^3^ Departamento de Fisiopatología, Hospital de Clínicas, Facultad de Medicina, Universidad de la República, Montevideo , Uruguay; ^4^ Advanced Bioimaging Unit, Institute Pasteur of Montevideo-Universidad de la República, Montevideo, Uruguay; ^5^ Department of Pharmacology, University of Texas Southwestern Medical Center, Dallas, TX, United States

**Keywords:** Arc, GUV, giant unilamellar vesicle, membrane remodeling, fluorescence, membrane budding

## Abstract

The activity-regulated cytoskeletal-associated protein (Arc, also known as Arg3.1) is an immediate early gene product induced by activity/experience and required for multiple modes of synaptic plasticity. Both long-term potentiation (LTP) and long-term depression (LTD) are impaired upon Arc deletion, as well as the ability to form long-term spatial, taste and fear memories. The best-characterized cellular function of Arc is enhancement of the endocytic internalization of AMPA receptors (AMPARs) in dendritic spines. Solution of the crystal structure of a C-terminal segment of Arc revealed a striking similarity to the capsid domain of HIV Gag. It was subsequently shown that Arc assembles into viral capsid-like structures that enclose Arc mRNA, are released into the extracellular space, and are internalized by neighboring cells. Thus, Arc is unique in participating in plasma membrane budding both into and out of the cell. In this report we study the interaction of Arc with membranes using giant unilamellar vesicles (GUVs). Using the fluorescent lipid probe LAURDAN, we find that Arc promotes the formation of smaller vesicles that penetrate into the GUV interior. Our results suggest that Arc induces negative membrane curvature and may therefore facilitate the formation of mRNA-containing extracellular vesicles from the plasma membrane.

## Introduction

The activity-regulated cytoskeleton-associated (Arc) protein is a major regulator of synaptic plasticity and long-term memory consolidation (Bramham et al., 2010; Zhang and Bramham, 2020). Changes in Arc expression influence the strength of individual synapses, during both long-term potentiation (LTP) (Rodriguez et al., 2005; Yilmaz-Rastoder et al., 2011) and long-term depression (LTD) (Park et al., 2008; Waung et al., 2008; Wall and Corrêa, 2018). Arc also modulates neuron-wide synaptic strength, as occurs during homeostatic plasticity (Jenks et al., 2017). Therefore, it is not surprising that inordinately low or high levels of Arc expression have been linked to a variety of cognitive disorders, including schizophrenia (Purcell et al., 2014), Alzheimer’s disease (Dickey et al., 2004; Lacor et al., 2004; Palop et al., 2005; Wegenast-Braun et al., 2009), Fragile X (Niere et al., 2012) and Angelmann (Greer et al., 2010) syndromes, as well as drug and alcohol abuse (Bramham et al., 2010). Mechanisms whereby Arc regulates synaptic strength are not entirely clear, although there is compelling evidence that it contributes to LTP by regulating the actin cytoskeleton (Messaoudi et al., 2007) and to LTD. by potentiating clathrin-mediated endocytosis of AMPA-type glutamate receptors (AMPARs) (Wall and Corrêa, 2018). The role for Arc in AMPAR endocytosis was supported by reports that Arc binds directly to three endocytic co-factors, dynamin (Chowdhury et al., 2006), endophilin (Chowdhury et al., 2006), and clathrin-adaptor protein 2 (DaSilva et al., 2016) as well as our finding that Arc stimulates dynamin self-assembly and GTPase activity (Byers et al., 2015).

Neuronal stimuli induce the rapid (within 5 min) transcription of the Arc gene (Ramírez-Amaya et al., 2005) and translocation (within 30 min) of its mRNA from the nucleus to the cytoplasm (Guzowski et al., 1999). In the cytoplasm, Arc mRNA incorporates into a large ribonucleoprotein (RNP) complex that is actively transported along the dendrite by a kinesin/microtubule-dependent mechanism (Kanai et al., 2004; Dynes and Steward, 2007). A component of this RNP complex is fragile X mental retardation protein (FMRP), which inhibits Arc mRNA translation. Neuronal activity induced, for example, by stimulation of metabotropic glutamate receptors (mGluRs), also triggers the rapid translation of a pool of pre-existing Arc mRNAs in dendrites (Link et al., 1995; Lyford et al., 1995; Steward et al., 1998).

Findings that the Arc gene may have a retroviral origin (Campillos et al., 2006) and that the C-terminal portion of Arc folds into a structure that bears a striking resemblance to the HIV-1 capsid domain (Zhang et al., 2015) led to the discovery of a different mechanism for Arc mRNA translocation. In 2018, two groups reported that Arc oligomers form virus-like particles that encapsulate Arc mRNA and that these particles are released from neurons as extracellular vesicles that are internalized by neighboring cells (Ashley et al., 2018; Pastuzyn et al., 2018). Thus, Arc apparently promotes both endocytosis and extracellular vesicle egress, processes associated with positive and negative membrane curvature, respectively. Although Arc binds directly to phospholipids (Barylko et al., 2018), its ability to deform membranes and to catalyze membrane scission had not been demonstrated. Here we report that purified Arc induces the formation of vesicles directed toward the lumen of giant unilamellar vesicles (GUVs), a process topologically equivalent to the budding of particles toward the exterior of the cell. We further show that Arc-mediated vesiculation occurs primarily from liquid-disordered domains of GUVs exhibiting liquid order/liquid disorder coexistence.

## Results

### Preparation of Nucleic Acid-free Arc

We previously reported that recombinant murine Arc elutes from gel filtration columns in several distinct but overlapping peaks, consistent with the presence of at least three low-order oligomeric species and one high-order (greater than 20 mer) species (Byers et al., 2015). A portion of the high-order species are likely to represent the relatively homogeneous population of ∼32 nm virus-like particles that were reported to encapsulate mRNA in a relatively non-selective manner (Ashley et al., 2018; Pastuzyn et al., 2018). RNA binding is important for high-order Arc oligomerization, as stripping of RNA from bacterially expressed human Arc reduces the A_260/280_ ratio from ∼1.0 to ∼0.68 and inhibits the formation of virus-like structures (Pastuzyn et al., 2018). Likewise, it was recently reported that Arc oligomerization increases upon addition of Arc mRNA (Eriksen et al., 2020).

**FIGURE 1:**
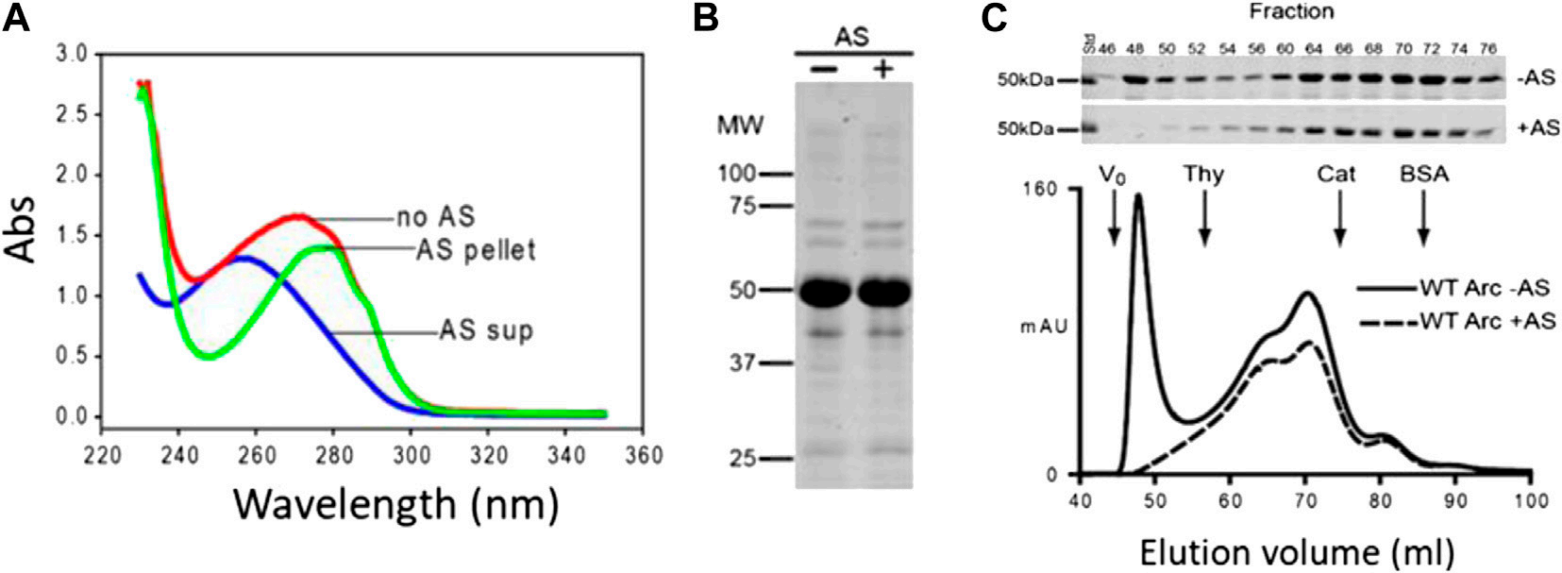
Removal of nucleic acids from Arc preparations by ammonium sulfate precipitation. **(A)** Absorption spectra of Arc preparations obtained without AS treatment (A_260/280_: 0.96), and of supernatant (diluted 20-fold; A_260/280_: 2.08) and pellet (A_260/280_: 0.57) obtained after AS precipitation (see Materials and Methods). Arc concentrations were 17.6 µM (no AS) and 16 µM (after AS). **(B)** Coomassie blue-stained gel of Arc prepared without and with AS treatment. **(C)** Superdex 200 elution profiles of Arc prepared with or without AS treatment. One ml of 60 µM (without AS) or 80 µM (with AS) Arc was loaded. Arrows designate standards: thyroglobulin (R_S_: 19 nm), catalase (R_S_: 5.2 nm), and BSA (R_S_: 3.67 nm). Chromatography was performed at 4°C.

In the present study, we sought to examine the membrane remodeling properties of nucleic acid-free Arc. Fortuitously, we found that the A_260/280_ of Arc decreased from ∼1.0 to ∼0.57 (as expected for a nucleotide-free protein) if bacterial lysates were precipitated with 35% ammonium sulfate (AS) prior to Arc purification (Figure 1A). The supernatant obtained after AS precipitation had an A_260/280_ of ∼2.0 (Figure 1A), a value indicative of pure RNA. AS treatment had no discernible effect on the electrophoretic patterns of our Arc preparations (Figure 1B). Importantly, Arc obtained from AS-precipitated bacterial lysates failed to display the high-order oligomeric peak that eluted near V_o_ but did not alter the elution positions of the internal peaks (Figure 1C). Thus, the Arc preparations that were analyzed in all GUV experiments presented below are nucleotide-free and do not form high-order oligomers (i.e., virus-like structures) in solution.

### Quantification of the Effect of Arc Addition on Giant Unilamellar Vesicles Morphology in the Fluid Phase

Arc contains α-helical N- and C-terminal domains connected by an intrinsically disordered linker region (Eriksen et al., 2020) (Supplementary Figure S1A). The C-terminal domain folds into a structure resembling the HIV Gag capsid domain (Zhang et al., 2015). The N-terminal domain contains determinants of self-assembly and lipid binding (Eriksen et al., 2020). We previously reported that Arc binds to small unilamellar vesicles formed from a brain lipid mixture approximately 5-fold more tightly than to vesicles formed from 100% phosphatidylcholine (PC). Because HIV-1 preferentially buds from membranes containing phosphatidylinositol 4,5-bisphosphate (PIP_2_), we tested whether phosphoinositides were responsible for the higher affinity of Arc to the more complex lipid mixtures. Co-sedimentation assays showed that Arc bound with similar affinities to pure PC liposomes and to PC liposomes containing 3 mol% phosphatidylinositol 4-phosphate (PI4P), phosphatidylinositol 3-phosphate (PI3P) or PIP_2_ (Supplementary Figure S1B). Thus, in contrast to HIV-Gag, the interaction of Arc with membranes is not appreciably enhanced by the presence of phosphoinositides. Hence, our initial studies of Arc-mediated effects on membrane morphology were conducted using GUVs generated from dioleoyl PC (DOPC) and labeled with LAURDAN as previously described (see, for example (Bagatolli et al., 2003; Pott et al., 2008; Malacrida and Gratton, 2018; Castro-Castillo et al., 2019)).

**FIGURE 2:**
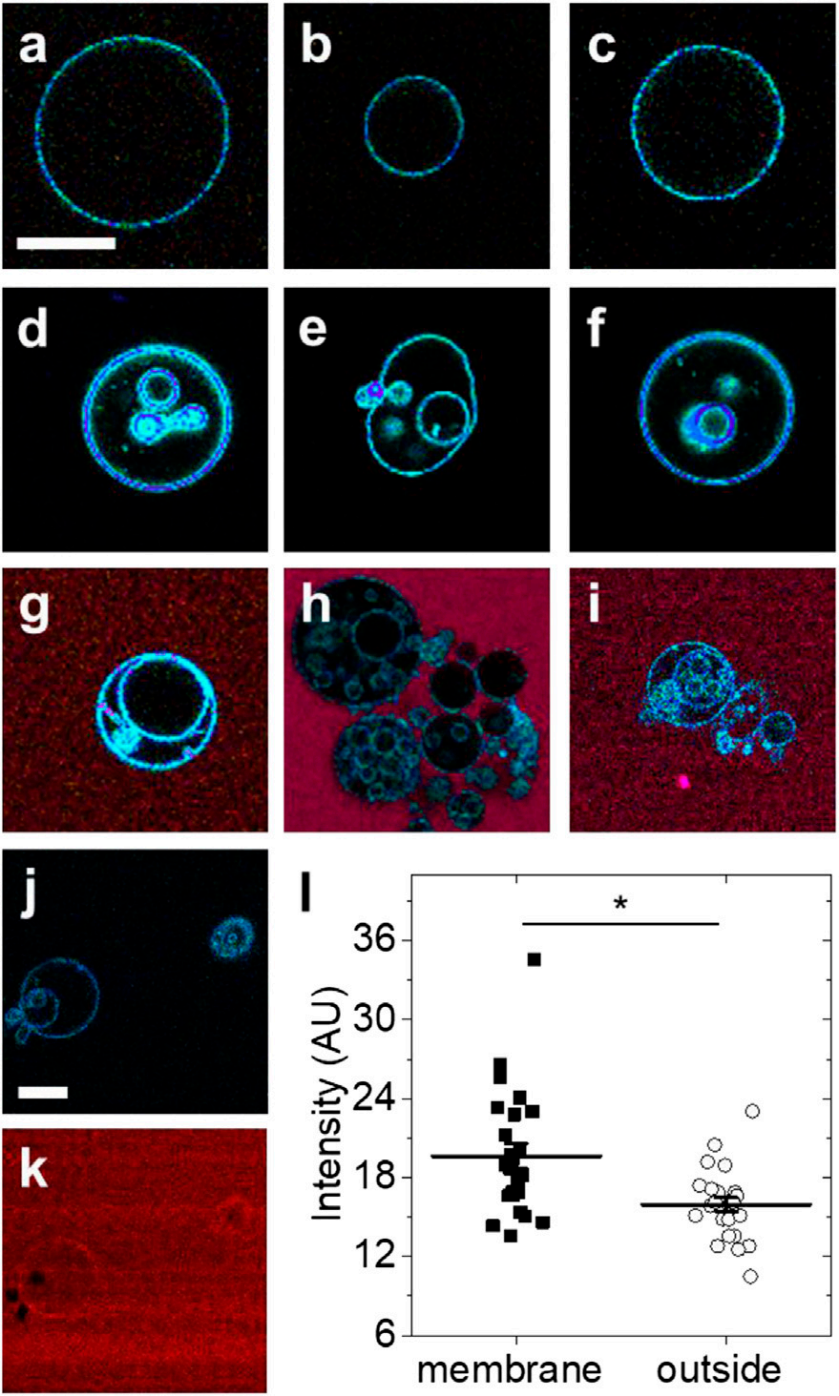
Exemplary fluorescence images of GUVs with and without protein addition **(A–C)** GUVs formed from DOPC and labeled with LAURDAN without protein addition **(D–F)** LAURDAN-labeled vesicles 2–60 min after addition of Arc **(G–I)** LAURDAN-labeled vesicles 2–60 min after addition of Arc-Alexa 594 **(J)** LAURDAN and **(K)** Arc-Alexa 594 fluorescence image of the same field of view **(L)** Fluorescence intensity of image pixels located on the membrane and outside the GUV shown in panel **(K)**; bars with whiskers indicate mean ± standard error (SE). Groups were compared with the Mann-Whitney *U* test (*) *p* < 0.05. Scale bars, 20 µm.

At room temperature, GUVs from DOPC lipids remain in the fluid phase. In the absence of Arc, GUVs from DOPC lipids typically did not show any internal structures. Exemplary LAURDAN fluorescence images are shown in Figures 2A–C. In contrast, dramatic alteration of the GUV structure was observed within 2–60 min after addition of Arc (2–9 µM) (Figures 2D–F). Many GUVs exhibited internal structures, either in the form of smaller vesicles or as irregularly shaped lipidic structures. To visualize Arc, we labeled the protein with Alexa 594 and repeated the experiment. Spectrally resolved images are shown in Figures 2G–I. As with unlabeled Arc, the formation of small internal vesicles was observed. Alexa 594 fluorescence remained strong in the solution outside the GUVs, indicating that labeled Arc had not accumulated significantly within the vesicles in less than 1 h. These observations suggest that Arc binds to the GUV membrane, which causes parts of the outer membrane to pinch off and to be released into the interior of the GUV as small vesicles. Binding of Arc to the GUV membrane is shown in Figures 2J,K, with panel **(J)** showing only the blue-green LAURDAN fluorescence and panel **(K)** showing only the red fluorescence from Alexa 594-labeled Arc. The difference in fluorescence intensity due to membrane-bound Arc and Arc in solution was quantified in Figure 2L and found to be significant, demonstrating that Arc accumulates on the GUV membrane. Arc binding to the GUV surface is more evident in Supplementary Figure S2A, in which labeled Arc has been added to GUVs while they are still attached to the platinum wire upon which they were electroformed.

**FIGURE 3:**
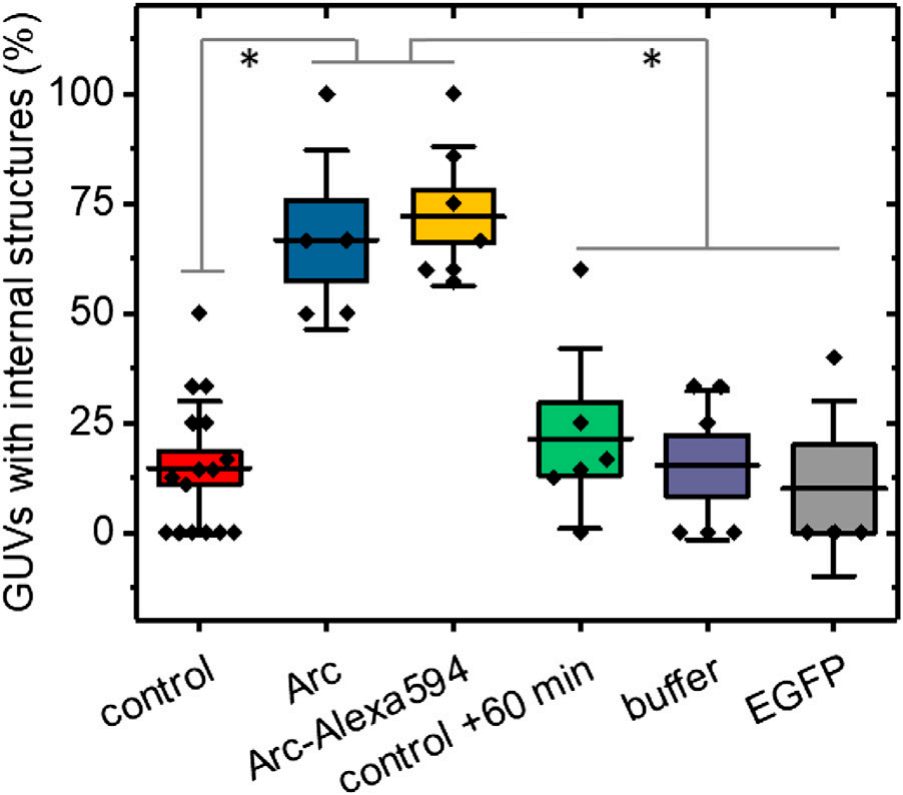
Percentages of GUVs showing internal structures with and without addition of Arc, horizontal lines represent the averages, boxes represent standard errors (± SE), and whiskers indicate standard deviations (± SD). No treatment controls were carried out at 0–20 min time (initial control) and at 60 + min. GUVs treated with Arc or Arc-Alexa594 (2–9 µM) as well as buffer and EGFP (5 µM) controls were examined from 2–60 min after treatment. Groups were compared with the Mann-Whitney *U* test, *p* values were < 0.01 (**) for Arc vs control +60 min, Arc vs buffer, and Arc vs EGFP, and < 0.001 (***) for Arc vs control, Arc-Alexa594 vs control, Arc-Alexa594 vs control + 60 min, Arc-Alexa594 vs buffer, and Arc-Alexa594 vs EGFP. Each data point represents the fraction of GUVs with internal structures averaged over 2–4 field of views (each 354 µm across). The total number of GUVs imaged was N = 217, comprised of N = 91 for the control, N = 22 for Arc, N = 36 for Arc-Alexa594, N = 32 for control + 60 min, N = 21 for buffer, and N = 15 for EGFP. For each group, at least three independent experiments were carried out.

To quantify the effect of Arc on the GUV structure, we repeated several experiments with 15–91 GUVs per group (Supplementary Table S1) and in each determined the percentages of GUVs with internal structures (Figure 3). The presence of internal lipids was observed in only 16.5% ± 2.4% (mean ± SE) of GUVs formed in the absence of Arc compared to 63.6% ± 10.5% of vesicles treated with unlabeled Arc. A similarly high fraction of GUVs with internal structures was observed for Arc labeled with Alexa 594. To ensure that time alone did not lead to the formation of internal vesicles, we re-imaged the control group after 60 + min and observed no significant changes. We also added the buffer solution without protein to exclude changes in osmolarity as a potential cause of lipid internalization. Finally, as a negative control and example of an inert protein, EGFP (5 µM) was shown not to induce internal vesiculation, and neither did Glutathione S-transferases (GST) protein (Supplementary Figure S2B).

### Effect of Arc on Giant Unilamellar Vesicles Formed from Complex Lipid Mixtures

**FIGURE 4:**
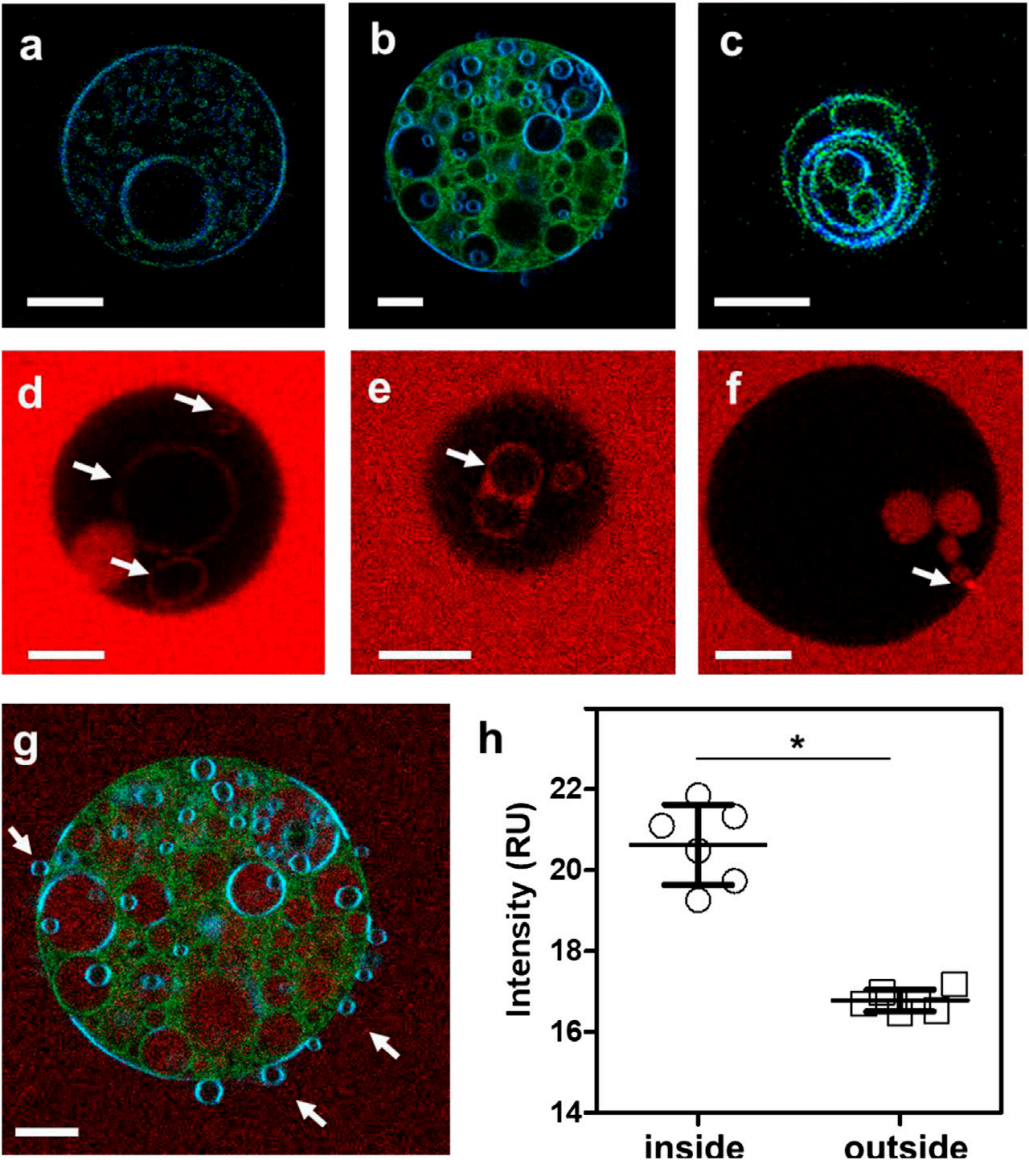
**(A–C)** Fluorescence intensity images of LAURDAN-labeled GUV composed of a ternary lipid mixture. Blue channel: 449/10 nm, green channel: 527/10 nm **(D–F)** Arc-Alexa647 fluorescence in the presence of GUVs. Arrows indicate Alexa 647-labeled Arc bound to the membranes of internal vesicles **(D,E)** and Arc protein populating the GUV interior **(F)**. **(G)** Red-green-blue overlay of LAURDAN and Alexa 647 fluorescence (red channel: 683/10 nm) of the GUV shown in panel **(B)**. **(H)** The Alexa 647 fluorescence was significantly higher in pixels inside compared to pixels outside the vesicle. Groups were compared with the Mann-Whitney *U* test (*) *p* < 0.05. Bars with whiskers indicate mean ± standard error (SE). Scale bars, 10 µm.

In a prior study, we showed that approximately 50% of Arc in mouse brain synaptosomes is resistant to extraction by cold Triton X-100 and is recovered in low buoyant density fractions following density gradient centrifugation (Barylko et al., 2018). These properties are consistent with the distribution of Arc to so-called “lipid rafts,” which are operationally defined as membrane microdomains enriched in cholesterol, sphingolipids, and saturated phospholipids (Pike, 2006). Lipid rafts have also been classified as liquid-ordered (L_o_) membrane domains, in contrast to the more fluid liquid-disordered (L_d_) domains which are enriched in unsaturated lipids (Bieberich, 2018). To test whether Arc-dependent vesicle formation occurs preferentially from L_d_ or L_o_ domains, LAURDAN-labeled GUVs were prepared from a ternary lipid mixture of DOPC, DPPC (1,2-dipalmitoyl-sn-glycero-3-phosphocholine) and cholesterol at a 1:1:1 ratio, which allows the separation of liquid phases (Ma et al., 2016). LAURDAN is one of the family of 2,6-naphthalene modified probes designed by Weber (Weber and Farris, 1979) to be sensitive to the polarity of its environment. LAURDAN’s emission is significantly red-shifted in disordered lipid environments (Parasassi et al., 1986). As shown in Figure 4A, the addition of unlabeled Arc to these GUVs induced the formation of internal vesicles, similar to those formed from DOPC vesicles. LAURDAN emission associated with these internal vesicles were generally greener in appearance (i.e., red-shifted), which is indicative of a more disordered lipid environment, relative to the bluer LAURDAN emission from the outer GUV lipid bilayer. After LAURDAN staining of the lipids, we added Alexa 647-labeled Arc to the GUVs. Examples of LAURDAN and Alexa 647 fluorescence images are shown in Figures 4A–C and Figures 4E,F, respectively. We note that for the GUV shown in Figure 4B, the Alexa 647 image overlay (Figure 4G) shows an accumulation of Arc inside the vesicle, confirmed by comparing the Alexa 647 fluorescence intensity inside and outside the GUV (Figure 4H). In rare cases, we also observed examples of positive membrane curvature (Figure 4G), arrows. The ability of Arc to protrude as well as to invaginate the GUV membrane may reflect the flexibility of the hinge region connecting its N- and C-terminal domains (Hallin et al., 2018) and may be relevant for its dual activities in extracellular vesicle budding and endocytosis.

## Discussion

The major goal of this study was to determine if Arc by itself can induce membrane curvature and budding, consistent with its putative role in extracellular vesicle release. Mechanisms of membrane vesiculation often utilize distinct sets of proteins to drive membrane deformation and scission, as illustrated by the process of clathrin-mediated endocytosis, which involves the sequential activities of curvature-inducing BAR domain proteins (e.g., endophilins) and membrane severing dynamins (Gallop et al., 2006). However, shallow insertion of amphipathic helices into one leaflet of the bilayer can promote membrane bending and severing by a single protein (Boucrot et al., 2012). For example, insertion of an amphipathic helix of the influenza virus M2 protein allows M2 alone to induce the release of M2-containing particles into the culture medium of transfected cells and into the lumen of GUVs (Rossman et al., 2010). Although Arc can bind directly to phospholipids (Barylko et al., 2018), its intrinsic ability to induce membrane curvature and/or scission, independently of associated proteins, has not hitherto been investigated. Our data indicate that Arc is also able to promote both the bending and severing of GUV membranes.

Due to their large size and low curvature, GUVs have been used to study the membrane remodeling activities of numerous proteins, including those involved in endocytosis and extracellular vesicle release. Introduction of endocytic proteins, e.g., dynamin (Hohendahl et al., 2017) and endophilin (Shi and Baumgart, 2015), to the outer surface of GUVs typically induces membrane extension and budding toward the medium, which is topologically equivalent to the cell cytosol. In contrast, proteins involved in the formation of extracellular vesicles, e.g., viral coat proteins (Bigalke et al., 2014) or elements of the ESCRT machinery (Wollert and Hurley, 2010), induce vesiculation toward the GUV lumen. The latter process has been referred to as “reverse topology” membrane scission. At physiologic pH and ionic strength, like HIV Gag, Arc can self-associate *in vitro* into multiple oligomeric species with oligomers ranging from monomeric (∼4 nm) to 30–40-mers (20–35 nm) (Byers et al., 2015). Interestingly, Arc has been implicated in both the internalization of endocytic vesicles carrying AMPA receptors and in the egress of virus-like particles carrying Arc mRNA into the extra-neuronal space (Ashley et al., 2018; Pastuzyn et al., 2018), suggesting that Arc can promote plasma membrane budding both into and out of the cell. In this study we show that Arc by itself promotes reverse topology scission.

Despite our previous observation that about 50% of Arc distributes to raft (L_o_-like) domains in neurons (Barylko et al., 2018), we found that Arc-induced vesicles preferentially internalize into GUVs from L_d_-like membrane domains. There are several other examples of proteins that associate with rafts in cells but distribute predominantly to L_d_ domains of GUVs, including α-synuclein (Fortin et al., 2004; Stöckl et al., 2008), SNARE proteins (Bacia et al., 2004), and HIV-Gag (Yandrapalli et al., 2016). Purified HIV-1 particles have raft-like lipid compositions, suggesting that HIV-Gag is sorted to, or induces the formation of, raft-like domains on the plasma membrane (Campbell et al., 2004; Brügger et al., 2006). However, a construct consisting of the N-terminal membrane-binding MA domain of HIV Gag linked to an artificial multimerization domain distributed almost exclusively to L_d_ (non-raft) domains of GUVs (Keller et al., 2013). Future studies will be aimed at identifying determinants within the Arc molecule that target it to cellular membranes, as well as post-translational regulations that regulate this targeting. We reported that Arc undergoes palmitoylation in neurons and that this modification is important for Arc-mediated control of synaptic weakening (Barylko et al., 2018). Palmitoylation tends to drive proteins to L_o_ domains (Levental et al., 2010). In these studies, we used bacterially-expressed Arc, which does not undergo palmitoylation. Therefore, it will be of interest to test whether palmitoylated Arc shows the same preference for L_d_ domains of GUVs exhibited by unpalmitoylated Arc.

## Materials and Methods

### Materials

pGST.parallel expression vectors were gifts of Dr. Hong Zhang (UTSW). His-MBP-TEV protease was a gift of Dr. Elizabeth Goldsmith (UTSW). Reagents for electrophoresis were from Bio-Rad (Hercules, CA, United States). Glutathione agarose was from Pierce (Dallas, TX, United States). Lipids were from Avanti Polar Lipids (Alabaster, AL, United States). Other reagents, buffers, and protease inhibitors and LAURDAN were from Sigma-Aldrich (St. Louis, MO, United States).

### Purification of Arc

The GST-Arc construct was expressed in *E. coli* Rosetta 2 cells. Cells were harvested after growing for 20 h at 16°C. GST-Arc was extracted from the bacterial pellet with solution A (20 mM HEPES, pH 8.0, 100 mM NaCl, 5 mM DTT, protease inhibitor cocktail consisting of 10 µg/ml each of N-p-tosyl-L-lysine chloromethyl ester, N-p-tosyl-L-arginine methyl ester, N-p-tosyl-L-lysine chloromethyl ketone, leupeptin, pepstatin A, and 0.2 mM phenylmethylsulfonyl fluoride (PMSF) and 0.05 mg/ml lysozyme). The extract was centrifuged at 100,000 x *g* for 1 h, Arc in the supernatant was precipitated with ammonium sulfate at 35% saturation, and the precipitates were resuspended and dialyzed against solution A to remove ammonium sulfate. To purify GST-Arc, solutions containing GST-Arc were incubated with glutathione resin for at least 5 h and the resin was first washed with solution A, then with solution A containing 0.2% Triton X-100, and finally with solution A containing 2 M NaCl (without detergent). Washed resin was incubated with His_6_-tagged TEV protease to release Arc (60:1 M ratio for 5 h). TEV was removed by incubation of Arc samples with His-tag purification resin (Roche). Purified Arc was dialyzed against solution B (20 mM HEPES, pH 7.5, 100 mM NaCl, 1 mM tris(2-carboxyethyl)phosphine (TCEP), and PMSF), aliquoted, frozen in liquid N_2_ and stored at –70°C.

### Size-Exclusion Chromatography (SEC)

Gel filtration chromatography of Arc was carried out by FPLC on a HiLoad 16/600 Superdex 200 column (GE Healthcare). Elution patterns were monitored by absorbance at 280 nm (A_280_). A_260_ was also measured and fractions were analyzed by SDS–PAGE and Coomassie blue staining. Calibration standards are listed in the legend to Figure 1.

### Liposome Binding Assay

Liposomes consisting of 100% brominated PC or 97% brominated PC plus 3% PI4P, PI3P, or PIP_2_ were prepared as described by Lee and Lemmon 2001 (Lee and Lemmon, 2001). Lipids were dissolved in chloroform and dried under a stream of nitrogen followed by overnight drying under vacuum. Dried lipids were resuspended in 20 mM HEPES (pH 7.4) and 100 mM NaCl, followed by 10 freeze/thaw cycles in liquid nitrogen and sonication in a bath sonicator (Laboratory Supplies Company, Model G112SPIT). Liposomes were then extruded 10 times through 0.1 μm filters using a Mini–Extruder (Avanti Polar Lipids). To remove aggregates, Arc solutions were centrifuged for 1 h at 314,000 x *g* at 25°C immediately before incubation with liposomes. Binding assays were carried out by incubating 3.5 μM Arc with liposomes for 15 min at 25°C in 20 mM HEPES (pH 7.4) and 100 mM NaCl. Samples were then centrifuged at 300,000 x *g* for 1 h at 25°C in a TL-100 tabletop ultracentrifuge. Supernatants were removed and pellets were resuspended in initial sample volumes. Equal volumes of supernatants and pellets were electrophoresed on SDS-polyacrylamide gels and proteins were visualized by Coomassie blue staining.

### Giant Unilamellar Vesicles Formation

GUVs were formed by electroformation (Angelova and Dimitrov, 1986) as previously reported (Bagatolli et al., 2003; Pott et al., 2008; Hedde et al., 2017; Castro-Castillo et al., 2019). Briefly, a set of Pt wires and a Teflon growth chamber were cleaned by sonication in ethanol and in chloroform for 60 min each. During sonication, 200 mM solutions of sucrose and glucose in demineralized water were carefully prepared to achieve equiosmolality between the two solutions. Lipid mixtures of either DOPC (1,2-dioleoyl-sn-glycero-3-phosphocholine) alone or DOPC, DPPC (1,2-dipalmitoyl-sn-glycero-3-phosphocholine) and Cholesterol (Avanti Polar Lipids, Alabaster, AL, United States) at a 1:1:1 volumetric ratio were prepared in chloroform at final concentrations of 0.3–0.4 mM of lipids. For fluorescence labeling, LAURDAN was added at a 1/200–1/500 dye-to-lipid ratio. All lipid samples were prepared in glass vials using Hamilton glass syringes cleaned with chloroform. After insertion of the Pt wires into the growth chamber, 3–4 µL of lipid solution (0.3 mM total lipids) was added to each wire, and the solvent was evaporated in a vacuum chamber for 30 min. After solvent evaporation, the camber was heated above 50°C to ensure that the lipids were above the melting temperature. To each of the three wells of the growth chamber, 350 µL of sucrose solution preheated to the same temperature was added. GUVs were formed by applying a 10 Hz sinusoidal electrical signal to the Pt wires with an amplitude of 2 V for 60–90 min. GUVs were released from the Pt wires by reduction of the frequency to 1 Hz for 10 min and transferred to 1.5 ml plastic tubes (Eppendorf, Hamburg, Germany). Glass bottom imaging chambers (Nunc Lab-Tek, ThermoFisher, Waltham, MA, United States) were prepared by coating with 1 mg/ml of BSA followed by the addition of 300 µL glucose solution to each well. GUVs were introduced to each chamber by adding 70 µL of solution. The higher density of the sucrose caused the GUVs to settle at the bottom of the imaging well.

### Fluorescence Imaging

GUV samples were imaged on a laser scanning microscope (LSM710, Zeiss, Oberkochen, Germany). Fluorescence was excited via a two photon process using a pulsed femtosecond Ti:Sapphire laser system (Mai Tai, Spectra Physics, Santa Clara, CA, United States) tuned to 780 nm for both LAURDAN and the Alexa dyes. The excitation beam was reflected off a 690-nm short pass dichroic mirror and fluorescence was detected with the LSM710 spectral detector in 32 channels in a wavelength range of 415–726 nm. Images of 256 × 256 pixels or 512 × 512 pixels were raster scanned with a pixel dwell time of 6.3 µs in regions of 35–354 µm. Each line was scanned four times and the signal averaged to improve the signal-to-noise. For consistency, images were generally taken at the GUV equatorial plane.

### Introduction of Fluorescent Proteins to Giant Unilamellar Vesicles

Arc was fluorescently labeled by reacting the protein with Alexa Fluor 594/647 NHS Ester (A20004, ThermoFisher, Waltham, MA, United States) overnight at 4°C. Labeled protein (∼1:1 labeling ratio) was separated from free dye using a Sephadex G-25 column (GE Healthcare, Marlborough, MA, United States). Arc-Alexa 594/647 was added to the GUV-containing imaging wells to a final protein concentration of 2–9 µM. EGFP was added to the GUVs at a final concentration of 5 µM. Fluorescence images were acquired within 90 min after protein addition.

### Image Analysis

Images of fluorescently labeled GUVs were quantified by visually determining the presence of internal lipid structures. If multiple substructures were present, only the largest was counted as one vesicle. Vesicles smaller than 5 µm were not considered in the analysis. Images were visualized with a linear rainbow color code from purple to deep red to visualize the 415–726 nm detection range of the 32 channel LSM710 spectral detector (Figures 2, 3) or by creating red-green-blue (RGB) image overlays (Figure 4) of the blue (449/10 nm), green (527/10 nm) and red channels (683/10 nm).

## Data Availability

The raw data supporting the conclusions of this article will be made available by the authors, without undue reservation.
