# Supplementary material for: Membrane Remodeling by Arc/Arg3.1
Source: Front Mol Biosci. 2021 Mar 8;8:630625. doi: 10.3389/fmolb.2021.630625 (PMC7982473; doi:10.3389/fmolb.2021.630625)
Supplement: Supplementary file 1 [file Presentation1.pdf]

Membrane Remodeling by Arc/Arg3.1

Per Niklas Hedde[1,2], Leonel Malacrida[3,4], Barbara Barylko[5], Derk D. Binns[5], Joseph P. Albanesi[5*], & David M. Jameson[1*]

[1]Department of Cell and Molecular Biology, University of Hawaii at Manoa, Honolulu, HI, USA.

[2]Laboratory for Fluorescence Dynamics, University of California, Irvine, CA, USA.

[3]Departamento de Fisiopatología, Hospital de Clínicas, Facultad de Medicina, Universidad de la República, Montevideo, Uruguay.

[4]Advanced Bioimaging Unit, Institut Pasteur of Montevideo-Universidad de la República, Montevideo, Uruguay.

[5]Department of Pharmacology, University of Texas Southwestern Medical Center, Dallas, Texas, USA.

[*]Correspondence should be addressed to Joseph.Albanesi@UTSouthwestern.edu and djameson@hawaii.edu

Supporting Information

Supplementary Figure 1.	Scheme showing the predicted structure of Arc. Liposome binding assay.
Supplementary Figure 2.	LAURDAN fluorescence in GUVs composed of a ternary lipid mixture.
Supplementary Table 1.	GUV data.

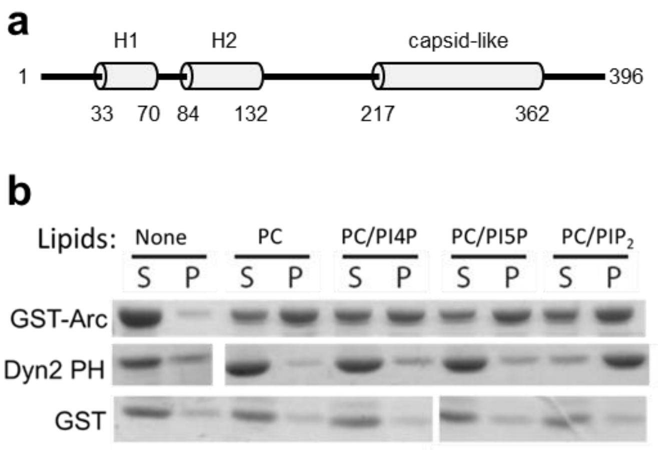

Supplementary Figure 1. (**a**) Scheme showing the predicted structure of Arc. Solid lines represent predicted disordered regions. H1 and H2 are predicted α-helical segments in the N-terminal domain. To date, only the structure of the largely α-helical capsid-like domain has been solved. (**b**) Liposome binding assay. GST-Arc was incubated with small unilamellar vesicles containing 100% PC or 97%PC and 3% PI4P, PI5P, or PI(4,5)P_2 (PIP_2). Supernatants (S) and pellets (P) were obtained after centrifugation, as described in Materials and Methods. The dynamin 2 (Dyn2) PH domain (which binds preferentially to PIP_2) and GST are shown as positive and negative controls, respectively.

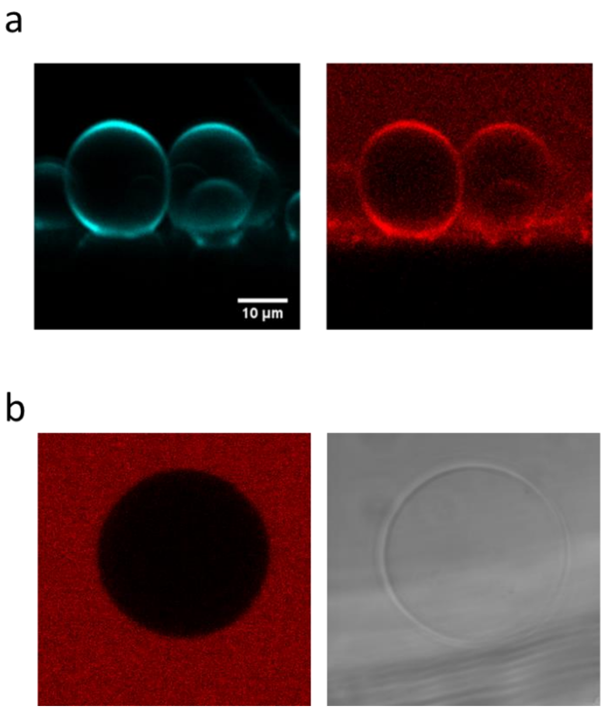

Supplementary Figure 2. (a) (left) LAURDAN fluorescence in GUVs composed of the ternary lipid mixture DOPC : DPPC : cholesterol (1:1:1 molar ratio), (right) Arc-Alexa 594 fluorescence in the same GUVs. **(b)** Absence of binding of GST-Alexa 594 to GUVs composed of the ternary lipid mixture.

Cohort	control	Arc	Arc-Alexa594	control +60 min	buffer	EGFP
1	4/8	3/6	3/5	1/7	1/3	2/5
2	1/7	2/3	3/5	1/8	0/4	0/5
3	1/9	2/4	2/3	0/2	0/3	0/1
4	0/7	4/6	5/5	1/6	1/3	0/4
5	1/4	3/3	6/7	3/5	1/4	
6	0/3		3/4	1/4	0/4	
7	1/7		4/7			
8	1/8					
9	3/9					
10	1/6					
11	0/5					
12	1/3					
13	0/4					
14	1/4					
15	0/3					
16	0/4					
**Total**	15/91	14/22	26/36	7/32	3/21	2/15

Supplementary Table 1. GUV data. Number of GUVs with internal structures/number of GUVs in each cohort as shown in Figure 3. Each cohort reports on the GUVs found in 2-4 field of views (each 354 µm across).
